# An easy adjustment of instrument settings (‘Peak MALDI’) improves identification of organisms by MALDI-ToF mass spectrometry

**DOI:** 10.1038/s41598-023-42328-2

**Published:** 2023-09-12

**Authors:** Christopher M. Nellessen, David B. Nehl

**Affiliations:** Department of Agriculture, Fisheries and Forestry, Sydney, Australia

**Keywords:** Mass spectrometry, Bacteria, Fungi, Entomology

## Abstract

Matrix-assisted laser desorption/ionization time-of-flight mass spectrometry (MALDI-ToF MS) is a mature technology with ‘auto-execute’ instrument settings and peak processing parameters tailored for rapid bacterial identification. Adoption for other organisms has been problematic, with optimisation efforts focusing on sample preparation. Using the Bruker MALDI Biotyper, we demonstrate ‘Peak MALDI’: easily-applied settings that immediately enhance sensitivity, improve spectrum quality, and increase identification confidence for any target, establishing its potential value for all MALDI-ToF MS systems.

## Introduction

Matrix-assisted laser desorption/ionization time-of-flight mass spectrometry (MALDI-ToF MS) has become a widespread technology, thanks in part to Federal Drug Administration approval for bacterial identification using the Bruker MALDI Biotyper and bioMérieux VITEK/Shimadzu MS systems in 2013^1,2^. Identification using MALDI-ToF MS systems involves comparing unknown sample results to reference mass spectra from verified samples. MALDI-ToF MS provides a rapid, effective identification process for bacteria that has predicated commercial development by a range of instrument manufacturers^3^, with its high-accuracy, and ease-of-use by those unfamiliar with mass spectrometry, underpinning its widespread acceptance.

While application of the technology has been explored for complex diagnostic targets, including fungi^2,4,5^, invertebrates^6–8^, plants^9^, and foodstuffs^10,11^, sensitivity and repeatability issues have been an impediment to broader adoption and commercialisation^12^. Efforts to improve results have largely focused on sample preparation^13–22^, and inconsistent outcomes and a lack of universality drive some to alternative mass spectrometry systems^23^.

Although sample preparation is important, the lack of substantial improvement is potentially based on an incomplete understanding of instrumental constraints. In MALDI-ToF MS, each laser shot produces a spectrum by generating ions that yield mass peaks with signal above the minimum signal-intensity threshold, set high by default to avoid conflation of diagnostic peaks with baseline noise. A final spectrum is constructed by averaging these laser-shot results. A potential lack of optimisation of these key acquisition parameters has been recognised in previous publications, with some spectral quality improvements noted with slight changes to both the laser shot totals and minimum threshold^24^. Lau et al. attempted to improve MALDI-ToF MS fungi identification by substantially lowering the minimum signal-intensity threshold^12^. Inconsistent performance resulted, as diagnostic peaks became harder to differentiate from noise despite enhanced sensitivity. A modification of data-acquisition settings to discriminate noise-obscured biomarkers present in unfractionated human serum, called ‘deep-MALDI’, was patented in 2013 for use with the MBT and involves using > 20,000 laser shots during data collection to produce superior spectra^25^. Later, the same researchers reported improved biomarker discrimination when applying even greater amounts of spectrum averaging, of 100,000,000 laser shots or more, in some of their published data^26^.

The noise-reduction benefits observed in deep-MALDI pairs well with the lower signal-acceptance settings proposed by Lau et al., with increased background noise offset by ensemble averaging. However, deep-MALDI and other approaches using tens of thousands of laser shots, greatly extend data collection time, due to an increased need to raster the laser to avoid impractical amounts of localised sample and matrix depletion. This makes such approaches impractical for routine identification. Therefore, we investigated the potential to combine a low minimum signal-acceptance threshold with increased numbers of laser shots to maximise signal detection and generate useful quality improvements to result spectra in a reasonable time frame.

## Results

Using a Bruker MALDI Biotyper sirius (MBT) mass spectrometer and systematic testing of three disparate organisms, we sought to determine whether an improvement to sensitivity, spectrum quality, and identification confidence could be achieved using a lower minimum-signal setting, randomized laser targeting, and increased total laser shots. Intending to improve upon previously explored sensitivity enhancements^12,24^, a minimum signal threshold of 3 arbitrary units was chosen for comparative evaluation against the default value of 600. Our chosen threshold of 3 arbitrary units maximises the MBT sensitivity, avoids potential peak-less data collection issues that may occur with a threshold of zero, and also relates by exactly an order of magnitude to the value of 30 arbitrary units used by Lau et al. This threshold was evaluated over a range of laser shot totals, comparable to those used during deep-MALDI, to assess if significant improvements are feasible.

Five replicate samples were prepared using up to three of the manufacturer’s protocols for each of the three organisms, with each preparation then applied to five MALDI target spots. Analysis of each spot employed default instrument auto-execute and default peak processing settings (minimum signal threshold of 600 arbitrary units and 240 total laser shots), followed by a set of analyses on the same spot featuring varied laser shot totals (240–10,000) paired with a constant minimum signal threshold of 3 arbitrary units. To accommodate potential sample or matrix depletion from higher laser shot totals and mitigate heterogeneity of sample and matrix on the test spot a *random walk* laser raster was also applied with the other altered settings, with laser shots per raster ranging from 1/10 to 1/20 of the total laser shot value. Effects of varied laser shots per raster were investigated separately (Supplementary Figs. 1, 2) to ensure that chosen values did not interfere with the comparative evaluation.

Comparative evaluation of results obtained using the default acquisition settings and the altered settings was facilitated using three quantifiable aspects of result spectra. These aspects relate to the following key qualitative measures: identification confidence, sensitivity, and spectrum quality. Identification confidence was assessed using the log score value of identity matches generated by the MBT software. Reference spectra from Bruker’s bacteria library (BDAL 2019) and Filamentous Fungi Library 3.0 (2019) were used to produce log score values for matches to the bacteria and fungi respectively, and the invertebrate was matched to a custom reference spectrum produced in-house. Sensitivity was quantified using the number of detected peaks. This value was limited to 100 to highlight the capability for these changes to work within the existing identification framework of the MBT software. Identification results for conventional MBT users would not be enhanced by exceeding 100 peaks, as this is the maximum peak number in Bruker’s reference spectra. Spectrum quality was quantified using the signal-to-noise ratio (S/N) of the highest-intensity peak. While the use of the highest S/N ratio would not be a reliable measure of spectral quality if considered in isolation, it facilitates straightforward comparison between spectra. Log score values, our knowledge of the corresponding reference spectra, and peak numbers provide context that evinces that the unevaluated peaks are of comparable S/N.

**Figure 1:**
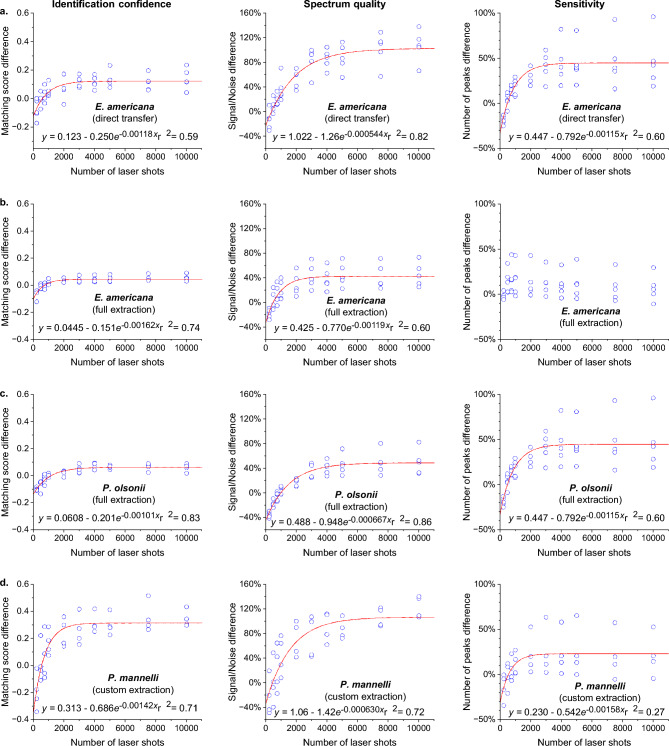
Increasing the number of laser shots enhanced Bruker MALDI Biotyper MALDI-ToF identification confidence (matching score), spectrum quality (signal-to-noise ratio), and sensitivity (number of peaks detected), when paired with a lower-than-default, minimum signal setting. Each data point is the difference between the average result from up to five duplicate test spots using default settings (240 laser shots) and the average from the same spots with results generated using our settings. For the graphs presenting the signal/noise and number of peaks, this difference value was converted to a percentage, relative to the corresponding default setting result, to enhance visual comparison of the setting results. Peak MALDI (minimum signal acceptance threshold of 3 paired with 2,000–10,000 shots) demonstrated superior results, while enabling sufficient sensitivity to collect data from all spots for all 1,000 tests.

**Figure 2:**
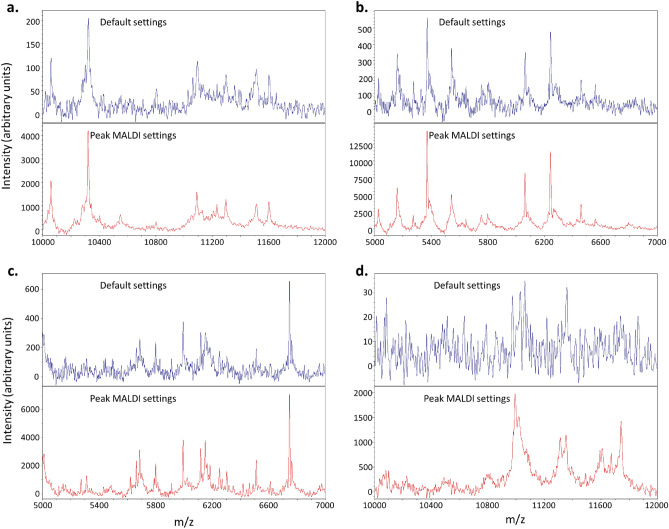
‘Peak MALDI’ (5000 laser shots) decreased spectrum noise and improved resolution of peaks in comparison to the default Bruker MALDI Biotyper settings; (**a**) *Ewingella americana* with *direct* preparation, (**b**) *E. americana*, and (**c**) *Penicillium olsonii* with *full extraction*, and (**d**) *Pholcus manueli*.

**Figure 3:**
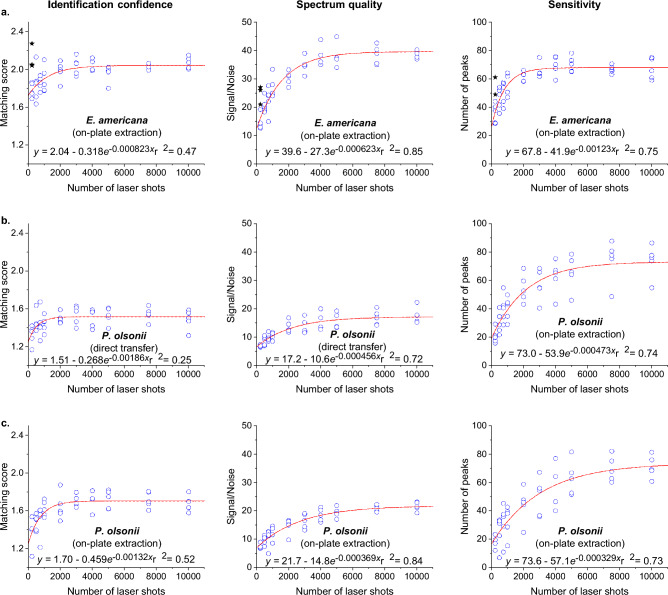
On-plate extraction of *E. americana*, direct transfer of *Penicillium olsonii*, and on-plate extraction *Penicillium olsonii* all resulted in the default settings regularly failing to detect ions above the minimum signal acceptance threshold while our altered settings were generally successful at detecting ions from these same test spots (Table 1). Successful default setting test spot results are displayed as black stars, and averaged replicates resulting from using our altered settings—each an average of up to five duplicate test spots—are displayed as blue circles. Our settings produced superior spectrum quality and sensitivity, and more consistent identification confidence, within the Peak MALDI range (2,000–10,000 laser shots).

First, we tested the bacterium *Ewingella americana* Grimont, using the three sample preparation methods recommended for the MBT: direct, on-plate extraction, and full extraction. Sensitivity, spectrum quality, and identification confidence were observed to increase with laser shots, and, above 2000 laser shots, consistently outperformed default setting results during identification, while providing visible sensitivity and quality improvements to resultant spectra (Figs. 1a,b, 2a,b and 3a). Increase rates were proportional to negative exponentials, consistent with ensemble averaging expectations, excepting low asymptotic boundaries caused by identification algorithm complexity and sensitivity’s diagnostic maximum. Signal detection and species matching rates from analyses of test spots performed using default settings, as well as analyses with our altered settings (for all 10 different laser shot totals combined), highlight the success of using the lowered minimum signal acceptance threshold of 3 arbitrary units in comparison to the default settings (Table 1). Our lowered minimum signal acceptance threshold setting enabled detection for all *direct* and *full extraction* tests, regardless of paired laser shot total, while default settings failed to collect any data in 4/25 *direct* and 2/25 *full extraction* tests, mitigating otherwise similar result quality (Table 1 and Fig. 1a,b). *On-plate extraction* featured a higher rate of data-collection failure, with our settings unsuccessful for 9/25 duplicate spots, equating to 90/250 tests, while default settings failed for 22/25 tests (Table 1; Fig. 3a). These high failure rates caused the data produced from this preparation method to be unsuitable for normalisation expressed as values of proportional difference, as presented in Fig. 1. Therefore, data for the altered settings and, when available, the default settings, were presented unnormalized as different symbols in Fig. 3.

**Table 1:** Signal-detection and reference spectrum matching for individual target-spot tests.

Reference spectrum	Preparation method	Default setting tests			All altered setting tests		
		Tests with peaks detected	Tests with no peaks detected	Correct match to reference spectrum	Tests with peaks detected	Tests with no peaks detected	Correct match to reference spectrum
*Ewingella americana*	Direct	21	4	84%	250	0	100%
	On-plate extraction	3	22	12%	160	90	62%
	Full extraction	23	2	92%	250	0	100%
*Penicillium olsonii*	Direct	0	25	–	250	0	87%
	On-plate extraction	0	25	–	236	14	82%
	Full extraction	14	11	56%	250	0	100%
*Pholcus manueli*	Custom	15	10	60%	250	0	97%

Next, we tested a culture of the fungus *Penicillium olsonii* Bainier & Sartory in the same fashion. Typically, direct sample preparation is considered unsuitable for fungi^27,28^, a conclusion supported by our results, as signal detection using default settings failed to collect data for all *direct* and *on-plate extraction* tests and for 11/25 *full extraction* tests (Table 1). Subsequently, only data from the full extraction preparation was normalised and expressed as values of proportional difference (Fig. 1c). The results from the other sample preparations of *P. olsonii*, which were only successful using the altered settings, are expressed as absolute values in Fig. 3. Notably, when using our altered minimum acceptance threshold with more than 2000 laser shots, peak signal was detected from all test spots, including the same test spots that had signal detection failures with lower laser shot totals. As observed with the bacterium, sensitivity, spectrum quality, and identification confidence increased with total laser shots for all three sample preparation methods (Figs. 1c and 3b–d).

Finally, we tested legs of the spider *Pholcus manueli* Gertsch to evaluate our instrument settings for a non-microbial identification target, using mechanical tissue disruption and a simplified chemical extraction to prepare samples^29^. Spectra were matched against a custom reference spectrum to obtain log score values. As before, sensitivity, spectrum quality, and identification confidence increased with laser shot total (Fig. 1d). There were substantial failures in signal detection with the default settings and none with any of the 10 different laser shot totals of our altered settings (Table 1).

## Discussion

Overall, when a minimum signal-intensity threshold of 3 arbitrary units was paired with 2,000 to 10,000 laser shots, sensitivity, spectrum quality, and identification confidence consistently outperformed default setting results for all tested organisms. The outcome of lowering the acceptance threshold is detection of lower-intensity peaks that are rejected under the default instrument settings, maximising the sensitivity of the test. Applying significantly more averaging, by using higher laser shot totals, decreases spectral noise potentially gained through this high-sensitivity approach, while increasing the intensity of consistently detected peaks—improving peak signal/noise ratios. The pairing of lower signal-intensity threshold with more laser shots makes lower-intensity peaks more distinguishable and increases peak numbers, which improves identification confidence. Peak-MALDI’s lower minimum signal acceptance threshold, increased number of laser rasters, and random laser raster pattern also compensate for heterogenous distribution of matrix and analyte. Our combined setting-adjustments enable successful data collection and identification of targets that may fail to produce any data with the default settings. Consequently, our settings provide mitigation of potential sample preparation-based detection failures and facilitates the use of more preparation approaches. The other benefits of detecting additional legitimate peaks include improved differentiation of taxa and broader capability to generate high-quality reference spectra from substrates that are relatively recalcitrant using the default settings, such as fungi and invertebrates. Test duration increased by less than 1 min/spot and outcomes were visually apparent as spectra with clearer, more abundant peaks (Fig. 2). Hence, we term the pairing of the successful laser shot range (2,000–10,000 shot total) and lower minimum signal-intensity threshold ‘Peak MALDI’. We noted diminishing improvements above 5,000 laser shots, and therefore recommend 5,000 laser shot Peak MALDI as a standard approach. We assume that a higher minimum signal acceptance threshold, paired with a lower number of laser shots than in Peak MALDI, could produce spectra of acceptable quality and diagnostic value when tailored for specific taxa groups, thus providing moderate time savings for high-throughput laboratory workflows. However, such bespoke settings risk losing the universal target utility and robustness afforded by the maximised sensitivity and randomised rastering of Peak MALDI. Consequently, Peak-MALDI is a logical tool that any laboratory using an MBT, or similar MALDI-ToF MS system, can easily apply to immediately improve results for any target.

## Methods

The settings edited in this example of Peak-MALDI correspond to those found on a Bruker Biotyper Sirius ROU MALDI-ToF system. It is assumed that similar changes could be made to other systems as appropriate to generate Peak-MALDI style settings, though the exact procedure to do so will vary by the UI and user access granted by the manufacturer’s MALDI control software. The method explained here includes the steps needed to enable these data collection changes on a MBT and a description of the methods performed for the systematic analysis of Peak-MALDI’s effect on results.

### AutoXecute method editing

FlexControl allows for AutoXecute method editing via the AutoXecute tab. Click the *Edit* button in this tab to begin the editing process. If the selected method in the top drop-down menu is the MBT_AutoX (the default method) *Save As* to generate a new Peak-MALDI method and rename the file appropriately.

Under the Evaluation tab of AutoXecute Method Editor window the “Peak Resolution must be higher than” should be changed from *400* to *500* to ensure the loss of resolution by this method through the extensive averaging is mitigated. The “Maximal Resolution __ times above threshold” in the Fuzzy Control section should be changed from *10* to *3* to help enable higher sensitivity through more data collection. The Processing Method under the Peak Evaluation must also be edited to enable higher sensitivity, and to do so the *Edit* button adjacent to this section must be clicked. In the Edit Processing Method window, the Mass List Find must be edited such that Minimum Intensity Threshold is changed from *600* to *3*. This enables much greater acceptance of signal by the instrument, enhancing sensitivity, but also increasing the amount of collected noise. The Peak Width should also be changed from *4* to *2* to, once again, ensure any loss of resolution by this method through the extensive averaging is mitigated, as peaks picked during analysis will be based on this expected resolution. Use the *Save As* button to save this new processing method under a new name, then exit this window.

The Movement tab should be changed so that a random walk is used, performed by ensuring the tick box next to *Random Walk* is checked. This ensures that a wide selection of the target spot is sampled, enabling the Peak-MALDI method to function without excessive consideration of sample depletion. After clicking this box, the “Shots at raster spot” should be changed to the appropriate laser shot per raster value for the Peak-MALDI method chosen. The higher this value the more ions will be produced during each raster, but excessively reducing the number of rasters by inflating this value can contribute to the false enhancement of a small population of detected ions due to sample inhomogeneity across the target spot (Supplementary Figs. 1, 2). Generally, the laser shot per raster value should vary between 1/10 to 1/20 of the total laser shot value. This ensures that there are enough portions of the target spot sampled to generate a good average of the ion signals without excessively decreasing the speed of collection.

The Accumulation tab should then be modified to ensure that in the Fuzzy Control section the “Sum up *240* satisfactory laser shots” is changed to the desired total laser shot total, which for Peak-MALDI varies between *2000* to *10,000* laser shots match and that the *40* next to “shot steps” is changed to match the value used in the Movement tab. Then ensure Dynamic Termination is set to Off in the Dynamic Termination section to enable a good sampling of each sample spot. Save the method file when changes are complete.

### Compass automatic data collection

To enable the newly generated method during routine, automated data collection, modifications to the Compass software used during automated data collection must be performed. In the Compass window, click “Settings”, represented by a gear icon in the top right of the home window. Select the “Identification” tab from the two choices on the left. In the now open Identification Profile window, choose the profile of the samples being tested with PEAK-MALDI and click the ellipse icon adjacent to the “AutoXecute method” option. From the file explorer pop-up select the newly generated method file. Click “OK” when finished to save the changes. This automated data collection setting can be returned to default data collection settings by reselecting the “MBT_AutoX” method from the choices of available methods in the “AutoXecute method” section of the settings after the data analysis using Peak-MALDI is complete, if desired.

### Sample preparation

Samples of the bacteria, fungi, and invertebrate were obtained by the Australian Department of Agriculture, Fisheries and Forestry during business operations. Cultures of *E. americana* and *P. olsonii* were used to prepare target spots using the each of the manufacturer’s recommended methods, namely the direct transfer, the direct transfer with formic acid overlay (termed on-plate extraction), and a full extraction procedure. Preparation for direct transfer involved using a sterile toothpick to scrape a colony of the target sample onto a MBT Biotarget plate (Bruker 1840375) followed by an overlay of 1 µL of 2.5 mg α-cyano-4-hydroxycinnamic acid (HCCA) matrix (Bruker CAS-No 28166–41-8) that was dissolved in 250 µL of Bruker standard solvent (Sigma-Aldrich 900666-100ML). Acid overlay of the direct transfer was performed in the same manner, but with an overlay of 1 µL of 70% formic acid (Sigma-Aldrich F0507-500ML) that was allowed to dry before the HCCA matrix overlay. The crude full extraction involved the use of a sterile plastic loop to collect sample material and transfer it to 300 µL of ultrapure water before deactivation with 700 µL of ethanol. The resulting sample was centrifuged at 13,000 rpm for two minutes, and the supernatant was removed. The process was repeated to ensure maximum removal of solution and allowed to air dry for five minutes. After drying, the sample was dissolved in 20 µL of formic acid followed by the addition of 20 µL of acetonitrile (Supelco 1.00029), centrifuged, and 1 µL was transferred to the corresponding target spots on the Biotarget plate. The legs of the *P. manueli* were prepared with a simplified variation of the full extraction, as modified from the method performed by Reeve et al*.*^29^, utilising a solution of 37.5% H_2_O, 2.5% TFA (Sigma-Aldrich 80457-10ML), 60% acetonitrile, and 12 mg/mL of ≥ 98% HCCA (Sigma C2020-25G). For the bacteria and fungi tests, a separate colony sample was targeted for each transfer to a set of five duplicate target spots, until five replicate sets of five duplicate spots were produced. For the *P. manueli* legs, 1 µL of supernatant from the crushed leg solution was spotted five times for each of the five legs used.

### Peak-MALDI testing and data analysis

The systematic testing of Peak-MALDI was performed on target plates prepared with spots of *E. americana*, *P. olsonii*, and legs from *P. manueli*. Data collection was performed sequentially, from the default method up to 10,000 laser shots for the first, third, and fifth replicate sets of spots. To control for the potential effect of sample depletion, the second and fourth replicate sets of spots were analysed in reverse, from 10,000 laser shots down to the default data collection settings. The number of laser shots at each raster spot were adjusted to reduce the data collection times at higher laser shot totals. The ratios, of total-laser-shots to laser-shots-per-raster-spot, used were 240:40, 500:50, 750:50, 1000:100, 2000:100, 3000:250, 4000:250, 5000:250, 7500:250, 10,000:250. Any changes caused by varying the number of laser shots per raster were not measured independently of the increased laser shot totals during systematic testing, and therefore possible effects on results were tested separately (Supplementary Figs. 1, 2). It was concluded that this parameter does not inflate results for the measured values, as despite an impact on signal intensity, relative intensities are conserved. Peak information was extracted from the data using FlexAnalysis and was analysed with OriginPro 2022 software. Default Bruker MBT processing parameters were used, which include smoothing with a Savitzky-Golay algorithm with a frame size of 25 Daltons, multipolygon baseline subtraction run twice with a 5 Dalton search window, and peak picking using a spectra differentiation algorithm with a maximum of 100 peaks. The data for each measured category was organized by collection group and laser shot total, resulting in five replicate sets of data, with each set containing five duplicate spot entries, repeated for each total laser shot value. The five duplicate spot results were averaged for each replicate set. The resulting averaged replicates were graphed using OriginPro 2023 software.

### Supplementary Information

Supplementary Figures.

## Data Availability

The AXE method files and the corresponding PRP processing file described by the paper are freely available from the authors upon request. All raw data generated and analysed during the current study are available from the corresponding author on reasonable request.
