# Supplementary material for: An easy adjustment of instrument settings (‘Peak MALDI’) improves identification of organisms by MALDI-ToF mass spectrometry
Source: Sci Rep. 2023 Sep 12;13:15018. doi: 10.1038/s41598-023-42328-2 (PMC10497524; doi:10.1038/s41598-023-42328-2)
Supplement: Supplementary file 1 — Supplementary Figures. [file 41598_2023_42328_MOESM1_ESM.pdf]

Supplementary Data Figures

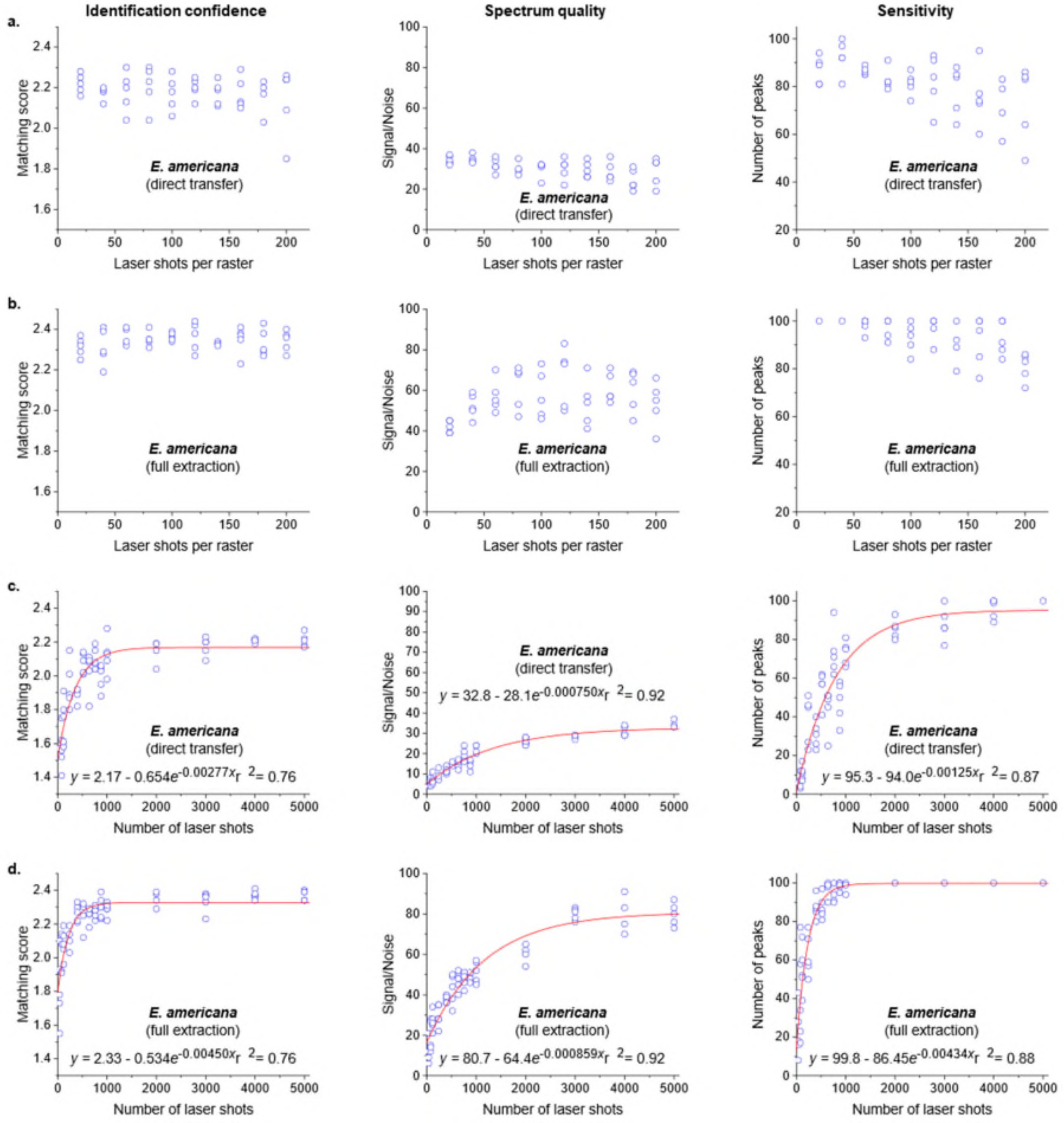

Supplementary Fig. 1: Two investigations, the first featuring constant laser shot totals (a, b) and the second featuring constant laser shots per raster spot (c, d), demonstrate that matching score, signal/noise, and number of peaks are not heavily impacted by the number of laser shots per raster. In the former, laser shots per raster were varied by 20 and results were nearly constant before the use of fewer rasters—signifying less averaging (more noise) and sampling (more inhomogeneity)—reduced values. The latter tests, with laser shot totals of 40, 80, 120, 240, 400, 520, 640, 760, 880, 1000, 2000, 3000, 4000, and 5000, featured results akin to main text investigations, reinforcing the conclusion that total laser shots impact results most. Therefore, focus should be on balancing low laser shots per raster spot's time-cost with the improvement in quality gained by their more extensive sampling and averaging.

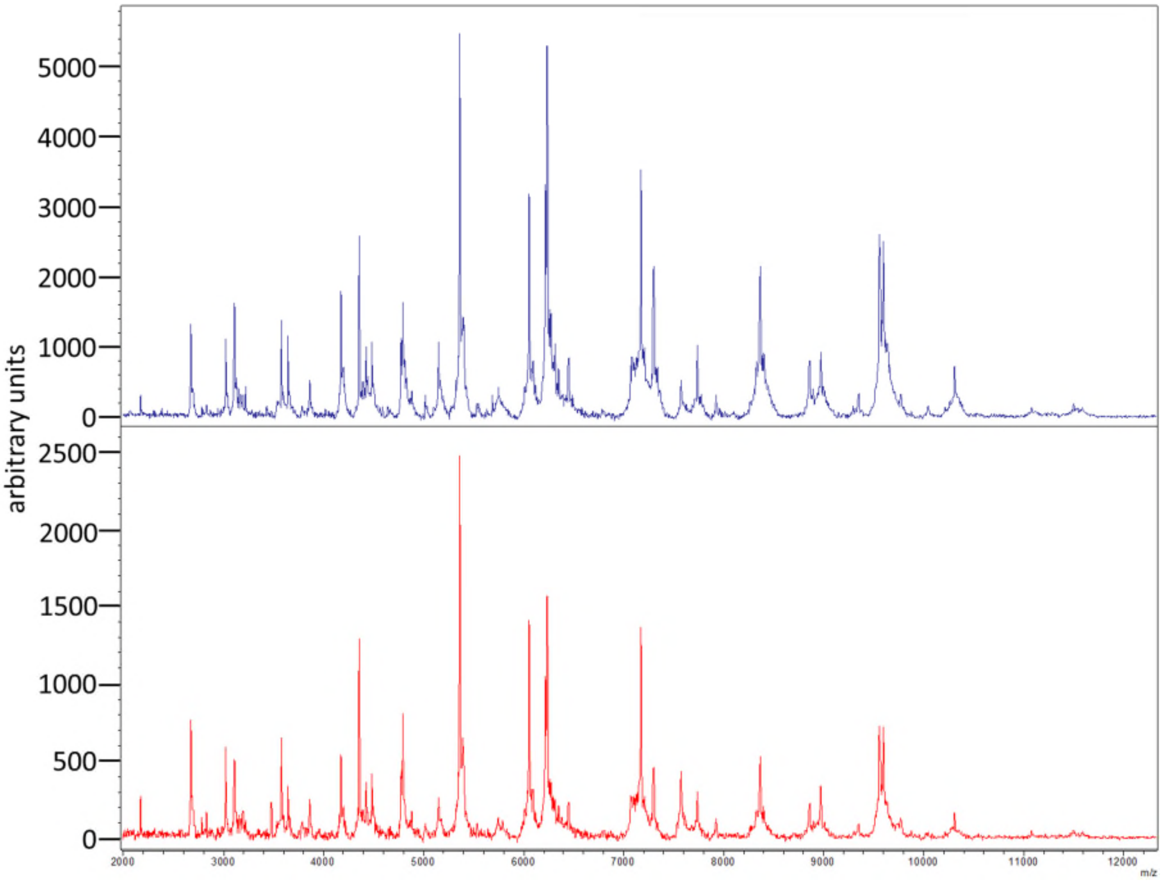

Supplementary Fig 2: More laser shots per raster increases peak intensity but does not improve the number of detected peaks or sensitivity of the spectrum, as this comparison of mass spectra produced from the same, full extraction prepared, *Ewingella americana* test spot, using 200 laser shots per raster (above) and 40 laser shots per raster (below), demonstrates. Furthermore, the Bruker scoring algorithm calculates an unchanged matching score between these spectra, because the relative peak intensities are generally conserved, reinforcing the conclusion that laser shots per raster has a low impact on results.
